# Anticipatory response before competition in Standardbred racehorses

**DOI:** 10.1371/journal.pone.0201691

**Published:** 2018-08-02

**Authors:** Zsófia Bohák, Andrea Harnos, Kinga Joó, Ottó Szenci, Levente Kovács

**Affiliations:** 1 MTA-SZIE Large Animal Clinical Research Group, Üllő, Dóra major, Hungary; 2 Department of Biomathematics and Informatics, University of Veterinary Medicine, Budapest, Hungary; 3 Institute of Animal Husbandry, Faculty of Agricultural and Environmental Science, Szent István University, Gödöllő, Hungary; Radboud Universiteit, NETHERLANDS

## Abstract

It is generally accepted that besides cortisol concentrations, parameters of heart rate variability (HRV) are appropriate indicators of stress in horses. The aim of this study was to determine anticipatory stress in eight Standardbred stallions participating in harness race. Cortisol and HRV responses to a mild exercise performed in training circumstances were compared to a maximal effort exercise performed in real trotting race conditions. Parameters of HRV reflecting vagal (root mean square of the successive differences, RMSSD) and sympathetic nervous system activity (ratio of the low and high frequency component, LF/HF) were recorded before warming up (baseline) and during exercise. Plasma cortisol concentrations were obtained for the following stages of the exercise: before warming up (baseline), after warming up, after the exercise has finished and after a 30-min recovery. Baseline LF/HF ratio was higher before the race compared to the pre-training values (12.0 ± 6.6 vs. 5.9 ± 4.5, *P =* 0.009), while RMSSD did not show such difference (34.8 ± 15.9 ms vs. 48.0 ± 30.5 ms, *P* = 0.96). Cortisol level was higher in the case of race for all samples compared to training (*P* = 0.012). There were no significant differences between plasma cortisol levels obtained for the subsequent stages of race. Horses in the present study showed anticipatory response before race as shown by differences in pre-training (97.3 ± 16.4 nmol/L) and pre-race cortisol levels (171.8 ± 18.7 nmol/L), respectively (*P* < 0.001). Pre-race HRV only partly confirmed this phenomenon.

## Introduction

The hormone cortisol is a versatile chemical regulator in every species. It plays a key role in daily homeostasis of the organism and regulated via the hypothalamic–pituitary–adrenal (HPA) axis. It is the primary hormone responsible for the stress response to physical and psychological challenges [1] Physical exertion results in the increase of the plasma cortisol in several species [2–6]. The exercise-induced response of the HPA axis is depending on the exercise intensity, duration [7], and the fitness level of the athlete [8,9]. Environment may also influence the cortisol response; besides physical challenges, psychological challenges can also affect the cortisol response, especially when participating to races [10,11].

As a noninvasive cardiovascular marker, heart rate variability (HRV) is often used for the assessment of stress and welfare in farm animals [12] as it reflects the changes of the sympatho-parasympathetic balance of the autnomic nervous system (ANS) in response to external stimuli [13]. Frequency-domain analysis of HRV provides relevant information about the short-term responses of the ANS in horses [14].

The relative little number of studies on equestrian training and competition focused on dressage or jumping horses and found increased post-competition plasma [15;16] and saliva cortisol levels [17] and decreased pre-race RMSSD [17]. In human athletes, emotional stress caused by racing is an important factor in the HPA response; however, no results are available on race-related cortisol secretion and HRV in racehorses. Therefore, we aimed to focus on the emotional effects of trotting race in Standardbred racehorses. We hypothesized higher cortisol levels and increased sympathetic tone before the race than before a usual training.

## Materials and methods

Eight trained Standardbred stallions between 3 and 4 years of age were used for the study that was specifically approved by the Ethics Committee of the University of Veterinary Medicine. The experiment was carried out between 2 and 4 April 2016 in Kincsem Park, Hungary, where trotting races are organized every Wednesday and Saturday. The daily routine and feeding do not differ between training and race days. However, on race days horses can hear the noise of transporters and the loudspeaker. The preparation of the horses occured on the same way on both experimental days, but the driver wore more colorful clothes on the day of race. Horses were kept in the same stable and trained by the same driver and horses were not transported during the experiment. During the period of study there were no significant differences in environmental temperature and humidity. The weekly workout plan of the experimental horses is described in Table 1.

**Table 1:** Weekly training schedule of the experimented horses.

Training schedule						
Monday	Tuesday	Wednesday	Thursday	Friday	Saturday	Sunday
Mild training	Mild training	Intensive exercise /race	Rest	Mild training	Intensive exercise /race	Rest

Measurements were performed in the same place under two different conditions: during training (mild exercise, n = 8) and during real trotting race (maximal effort, n = 8). The distance covered during training and race was 8,000–9,000 and 2,300 m, respectively. All the exercises were performed between 10:00 AM and 11:45 AM and lasted approximately for 48–55 min at all. Blood samples were obtained by venipuncture from the jugular vein (S-Monovette 7.5-mL Z tubes; Sarstedt, Nümbrecht-Rommelsdorf, Germany) according to the same pattern for the training and racing sessions considering the diurnal rhythm of cortisol secretion [18] as follows: S0; pre-race, 5 min before warming up, S1; after warming up (17–20 min after S0), S2; immediately after the intensive stage of the exercise (15–20 min after S1 depending on the type of work) and S3; after a 30-min recovery (30 min after the exercise has finished). Blood was centrifuged at 2,000 *g* for 10 min, 15 min within the actual sampling has completed.

Cortisol assay was performed in duplicate by direct radioimmunoassay with a HPLC preparation of cortisol-3-corticosterone methyloxidase, coupled with 2-[^125^I] iodohistamine as tracer for specific antibodies raised against cortisol-3-CMO-BSA [19].

For the measurement of interbeat intervals (IBI), horses were equipped with a Polar Equine RS800CX multi device and a Polar H2 sensor (Polar Ltd., New York, USA). After a 1-h habituation period to the equipment, IBIs were recorded before the exercise for one hour between 07:00 and 08:00 AM at rest, and during the exercise. Equal length of 5-min continuous IBI samples were used for HRV analysis. The Kubios HRV software (version 2.2, Biomedical Signal Analysis Group, Department of Applied Physics, University of Kuopio, Finland) was used for the analysis of IBI data. Type 1 errors (QRS detected prematurely when in fact a sinus-conducted wave has not occurred) and Type 2 errors (failing to detect an R wave that is present) were removed from the dataset as well as irregular sinus rhythms. Using the custom filter of the program, every IBI that differed more than 30% from the previous one, was replaced by an interpolated value calculated from the differences between the previous and the next accepted IBI. Slow nonstationary trend components were removed by using the ‘smoothness priors’ based detrending approach with λ = 1000 and f_c_ = 0.029 Hz. For representing vagal regulatory activity, the RMSSD was computed, whereas LF/HF was presented to assess the sympathovagal balance.

For hypothesis testing, a linear mixed model was fit by the Restricted Maximum Likelihood method [20]. Cortisol and parameters of HRV were the response, while the type of work (training or race) and the sampling occasion in interaction were the explanatory variables. The dependence between measurements from the same horse was considered by modelling the within-horse error variance–covariance matrix. Model effects were tested together based on their *t*-values. For multiple comparisons, we tested contrasts. Tukey’s method was applied to avoid the accumulation of Type I error. Significance was set at the level of 0.05. The analysis was carried out using the R 2.15.1 statistical software [21].

## Results

**Fig 1:**
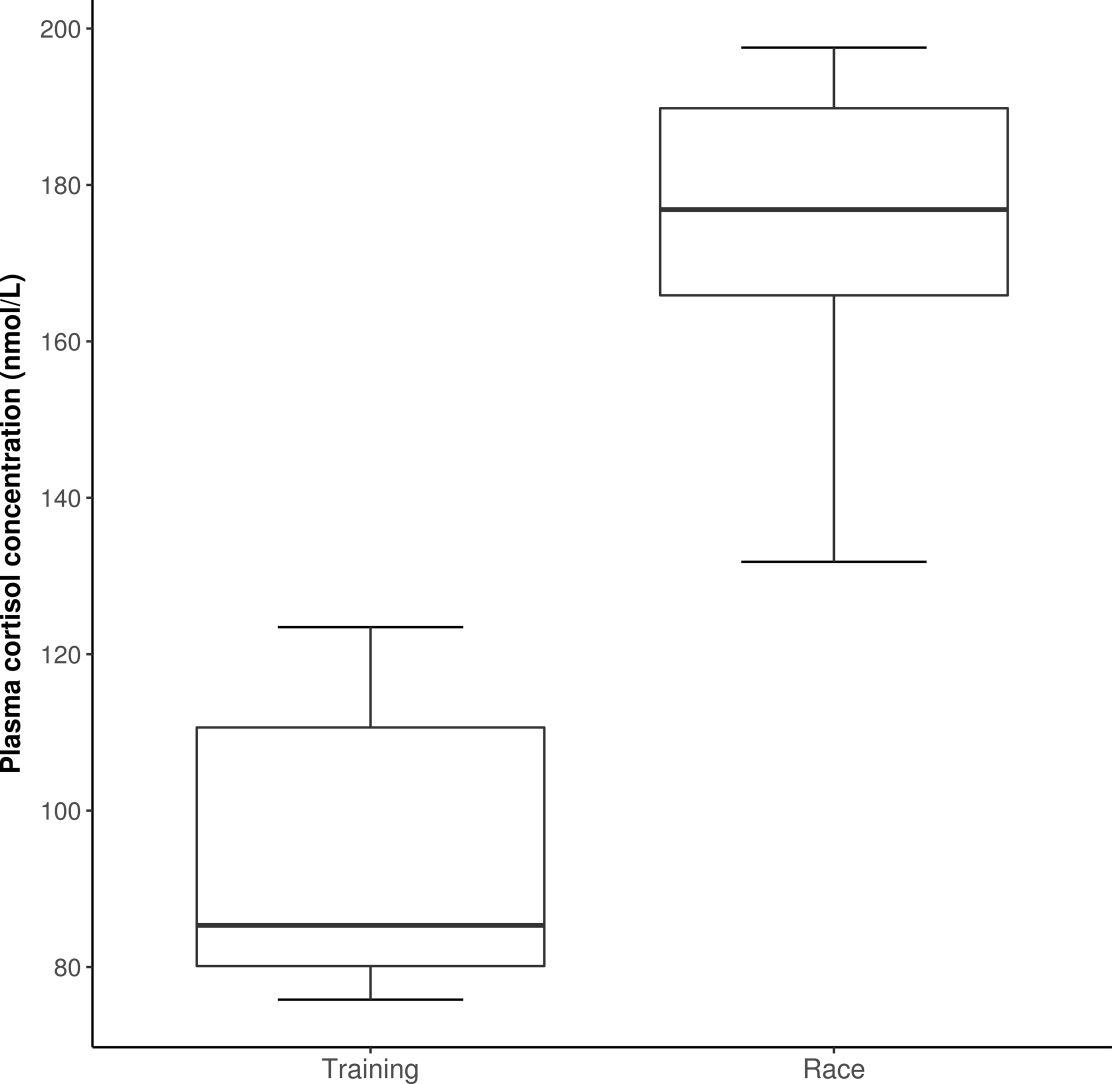
Plasma cortisol levels (median, 25 and 75 percentiles, min/max) of Standardbred racehorses (n = 8) before training and trotting race. Samples were taken between 10:00 and 10:30 AM, 5 min before warming up.

Baseline LF/HF ratio was higher (12.0 ± 6.6 vs. 5.9 ± 4.5, *P =* 0.009), while baseline RMSSD did not show significant difference (34.8 ± 15.9 ms vs. 48.0 ± 30.5 ms, *P =* 0.96) when pre-race and pre-training values were compared. Pre-race cortisol levels on training and race days were 97.3 ± 16.4 nmol/L and 171.8 ± 18.7 nmol/L (means ± SD), respectively (*P* < 0.001, **Fig 1**).

**Fig 2:**
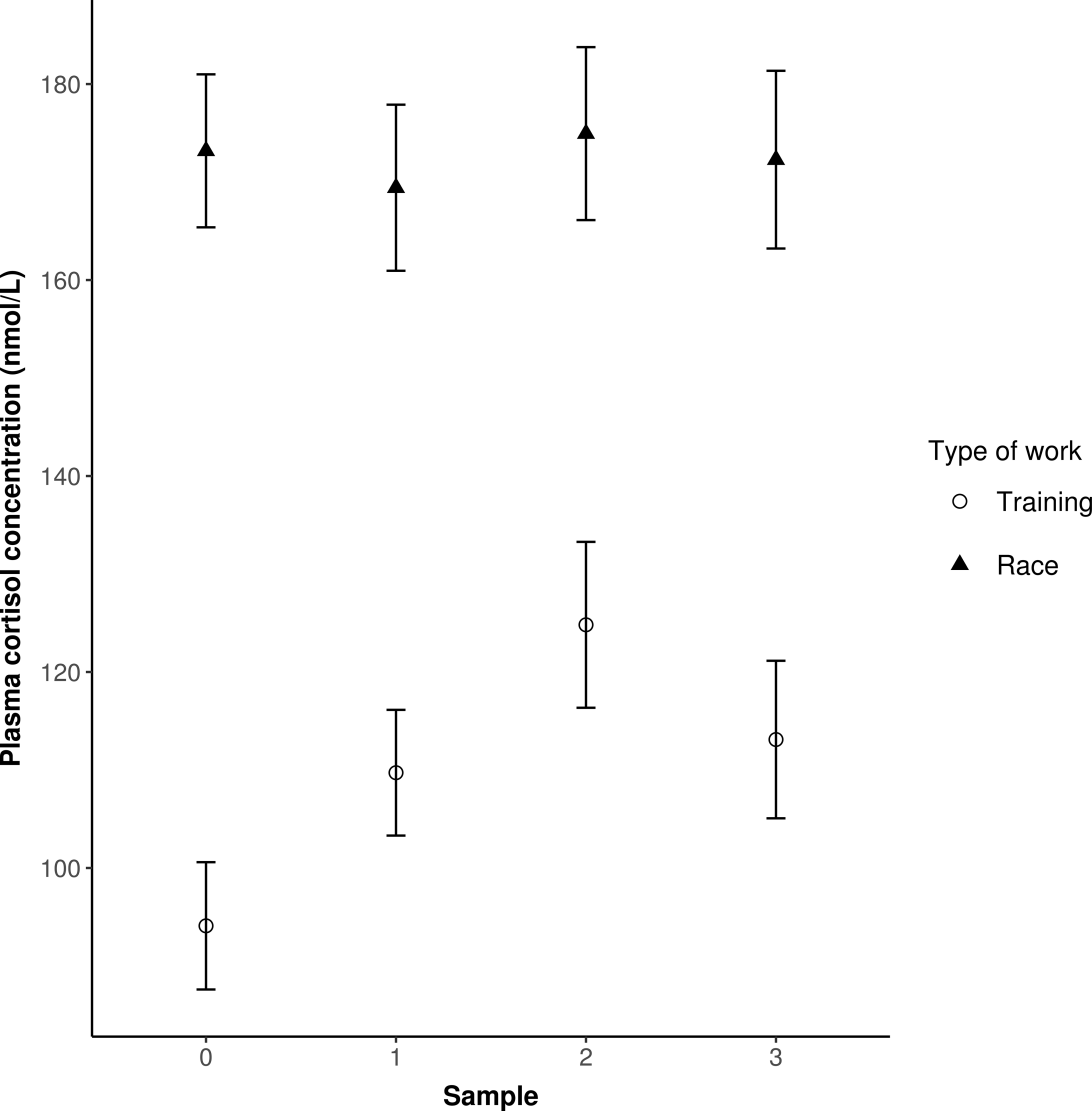
Plasma cortisol levels of Standardbred racehorses (n = 8) before and after training and trotting race (means ± SEM). 0: Sample 0 = before warming up (baseline), 1: Sample 1 = afer warming up, 2: Sample 2 = immediately after the end of exercise, 3: Sample 3 = after a 30-min recovery. The distance covered during training and race was 8,000–9,000 and 2,300 m, respectively. The time lag between the first and the last sample ranged between 52 and 60 min.

According to the linear mixed model, both the type of work and the time of sampling had a significant effect on the cortisol level (*P* < 0.001 and *P* = 0.012, respectively). Cortisol level was higher in the case of race at every sampling (**Fig 2**).

## Discussion

In this paper, plasma cortisol levels and HRV of Standardbred racehorses were assessed before and after training and trotting race. Since emotional effects of trotting race might influence basal physiology of horses, higher HPA activity and increased sympathetic tone before the race than before training were hypothesized. Our results supported the hypothesis. Elevated plasma cortisol concentrations and increased LF/HF ratio before the race than before a usual training suggest that horses might be exposed to anticipatory stress. However, the unchanged pre-race RMSSD did not confirm this assumption. A recent paper examined anticipatory responses in horses prepared before dressage and show jumping during a 3-day championship [17]. RMSSD and heart rate continuously increased during the pre-competition preparation, while pre-competition salivary cortisol did not change compared to previously measured basal cortisol concentrations. The lack of the pre-competition salivary cortisol increase indicate that these horses are less anticipated before competitions compared to racehorses used in the present investigation. The atmosphere and the expected performance are also less different from everyday workouts, therefore, decreased RMSSD may be not the result of the forthcoming race but rather only the usual effect of saddling in the authors’ study.

The present study describes cortisol and HRV differences between training and race. It has been shown that human intervention and the forthcoming physical exercise mean stress for the horse [2–6]. Preparation probably affected baseline RMSSD in our study as well, but this effect was not significantly stronger before the race compared to the training. However, the higher pre-race LF/HF ratio and cortisol level suggest a more marked sympathetic overweight and presumable extra stress before race compared to the training day. During the experimental period, horses were kept in stables near the racing track. They were exposed to the atmosphere of the race in every Wednesday and Saturday. It cannot be excluded that these effects affect HPA function not only in the experimental animals, but of all horses kept in these stables, even when they have not been entered to the given race. The elevated pre-exercise sympathetic tone before race may reflect the totality of many impacts, like the environment, the forthcoming exercise and the associated competition stress.

After the relative high pre-race cortisol level, no further cortisol rise was found during the race, while on training days, significant increase was observed from baseline. It contradicts to the findings of von Lewinski et al. [22], who detected similar increase in cortisol level of sport horses during training and public performance. It is a limitation of our study that difference in distances between training and race sessions taken by athletes might have influenced HPA responses at S2 and S3 samplings. Nevertheless, the lack of a significant cortisol increase during the race can be explained rather with the excessively high basal values than with the shorter distance. The high pre-race cortisol level may have caused a negative feedback to the HPA axis and inhibited further ACTH release during the exertion. In other investigations, where also positive cortisol response to maximal effort was found, the basal cortisol level was unaltered, and cortisol started to increase from a much lower value compared to our study [5,6, 23–25]. To study performance-related increases in cortisol levels it would be important to exclude the effects of race atmosphere. However, maximal performance outside the competition can be achieved only with unusually powerful encouragement of the driver which also would cause additional stress for the horse.

Our HRV data recorded during exercise were uninterpretable for several reasons. The length of IBI data recorded during race sessions did not allow us the proper analysis of HRV during exercise. Moreover, the displacement of the electrodes under the harness saddle was unavoidable at this high speed (45–50 km/h). It was shown that HRV above heart rate of 120–130/min is predominantly determined by non-neural mechanisms [26] during a mild intensive exercise, therefore, failure of recording valid IBI data could not be considered as a serious shortcoming of our study.

### Conclusions

Pre-race plasma cortisol concentrations and sympathetic tone-related HRV reflected anticipatory response in racehorses before trotting race. Vagal tone related HRV did not confirmed this phenomenon. The lack of a significant cortisol increase during race might have resulted from the excessively high basal values. The comparison of plasma cortisol levels measured during training and race seem to have no relevance in this experiment, because the work effort could not be precisely determined due to various environmental and emotional effects that should be further investigated in physiological studies on equine performance.
