# Correction: Anticipatory response before competition in Standardbred racehorses

**DOI:** 10.1371/journal.pone.0208521

**Published:** 2018-11-29

**Authors:** Zsófia Bohák, Andrea Harnos, Kinga Joó, Ottó Szenci, Levente Kovács

The following information is missing from the Funding section: This research was supported by the 17896-4/2018/FEKUTSTRAT grant of the Hungarian Ministry of Human Capacities.
